# Metabolic reprogramming in inflammaging and aging in T cells

**DOI:** 10.1093/lifemeta/load028

**Published:** 2023-06-28

**Authors:** Alessio Bevilacqua, Ping-Chih Ho, Fabien Franco

**Affiliations:** Department of Fundamental Oncology, University of Lausanne, 1007 Lausanne, Switzerland; Ludwig Institute for Cancer Research, University of Lausanne, 1066 Epalinges, Switzerland; Department of Fundamental Oncology, University of Lausanne, 1007 Lausanne, Switzerland; Ludwig Institute for Cancer Research, University of Lausanne, 1066 Epalinges, Switzerland; Department of Fundamental Oncology, University of Lausanne, 1007 Lausanne, Switzerland; Ludwig Institute for Cancer Research, University of Lausanne, 1066 Epalinges, Switzerland

**Keywords:** immunometabolism, inflammaging, T cells

## Abstract

Aging represents an emerging challenge for public health due to the declined immune responses against pathogens, weakened vaccination efficacy, and disturbed tissue homeostasis. Metabolic alterations in cellular and systemic levels are also known to be cardinal features of aging. Moreover, cellular metabolism has emerged to provide regulations to guide immune cell behavior via modulations on signaling cascades and epigenetic landscape, and the aberrant aging process in immune cells can lead to inflammaging, a chronic and low-grade inflammation that facilitates aging by perturbing homeostasis in tissues and organs. Here, we review how the metabolic program in T cells is influenced by the aging process and how aged T cells modulate inflammaging. In addition, we discuss the potential approaches to reverse or ameliorate aging by rewiring the metabolic programming of immune cells.

## Introduction

CD8^+^ T cells are a central component of the adaptive immune system to protect the body against exogenous pathogens and tumors. Upon recognition of peptide-major histocompatibility complex I by T-cell receptor (TCR), naïve T cells can differentiate into effector T cells to fight against the threat and form different memory subsets to ensure long-term protection. Emerging evidence shows that the different subsets of T cells, during the infection course, require distinct metabolic profiles to support their specific energetic demands but also to regulate their differentiation and epigenetic programming [1, 2]. Quiescent naïve T cells rely on mitochondrial oxidative phosphorylation (OXPHOS) to maintain themselves in secondary lymphoid organs [3]. Upon activation, the bioenergetic needs of T cells are higher to support their proliferation and effector function to fight against the pathogen. TCR signaling will engage the Rapamycin (mTOR) pathway, and T cells will upregulate their nutrient uptake and increase aerobic glycolysis, glutaminolysis, and mitochondrial ­biogenesis [4]. Memory T cells have been shown to reduce the mammalian target of the mTOR pathway and aerobic glycolysis. They display a more quiescent metabolic phenotype and rely on fatty acid oxidation (FAO) and mitochondrial metabolism to support their persistence in the organism [5, 6]. Memory T cells will also adapt their mitochondrial fitness by promoting mitochondrial biogenesis and engaging its inner membrane fusion mediated by Optic Atrophy 1 to ensure mitochondrial quality and cristae maintenance [7, 8]. This improved mitochondrial fitness and FAO reliance promote the spare respiratory capacity of memory T cells, which is their ability to produce extra energy upon high energetic demand. This parameter is not only important for their differentiation and survival but also for their rapid and efficient recall ability upon reencountering pathogens [9]. Consequently, metabolic reprogramming is considered a key element in CD8^+^ T cells’ function and lineage. However, during aging, the immune system progressively loses its ability to protect the host against diseases. For instance, infections in elderlies have higher chances to be fatal and cancer is more frequent in the elderly population. Recent studies observed metabolic shifts in aging T cells and those dysregulations directly contributed to age-related T-cell dysfunction. In this context, understanding how T-cell responses are altered during aging will provide critical insights into how elderly populations can be better protected against pathogenic threats. In this review, we will cover current knowledge on how metabolic pathways fail and are dysregulated upon aging, thus limiting T-cell function and differentiation. We will also discuss current and prospective strategies to target impaired metabolism in aged individuals.

## Hallmarks of immune system aging

**Figure 1:**
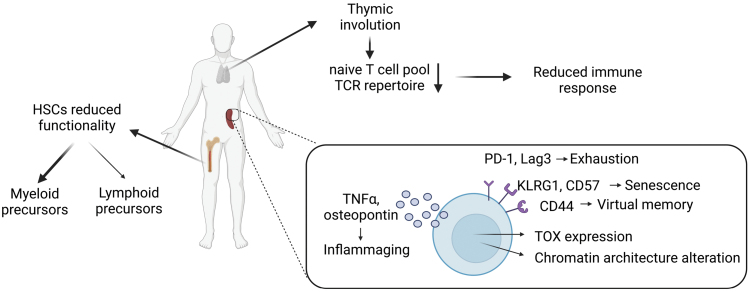
During the aging process, clonal expansion of HSCs skews toward the generation of myeloid precursors and reduces the output of newly formed lymphoid precursors. Thymic involution, which is the shrinkage in size of the thymus, also contributes to the loss of the T-cell compartment renewal in aged individuals. Aged T cells also display altered chromatin landscape, accumulate DNA damages, and acquire some exhaustion characteristics, including upregulated expression of TOX, PD-1, and Lag3, and senescence markers such as KLRG1 or CD57. They also secrete proinflammatory cytokines like TNF and osteopontin.

Hematopoietic stem cells (HSCs) are responsible for the production of all our circulating immune cells including lymphocytes and myeloid cells. During aging, the decline of pluripotency in HSCs leads to a biased generation toward increased myeloid cell output (Fig. 1) [10]. This process is partly due to the alterations in the transcriptional program favoring the myeloid cell compartment [11, 12]. More precisely, several cell-intrinsic factors have been shown to play a role in HSC aging: replicative stress, DNA damage responses, epigenetic landscape changes, and metabolic stress [12]. Strikingly, aging is associated with the increased development of myeloid leukemia, which further highlights the importance that cellular properties of HSCs can be influenced via extrinsic signals [13]. Interestingly, the environment provided by stromal cells in the bone marrow also drives phenotypic changes in HSCs during aging, suggesting that the functional alterations of HSCs are not strictly intrinsic [14–16]. The importance of intrinsic versus extrinsic causes remains unclear and still needs to be addressed. Myeloid cells’ functionality during aging is also altered [17]. For example, neutrophils of aged individuals display reduced phagocytic activity [18] and show impaired migration which can result in organ damage in mice [19, 20]. In addition, macrophage functionality is also impaired with aging with reduced responsiveness to stimuli of Toll-like receptors [21–23].

At the levels of T lymphocytes, both CD8^+^ and CD4^+^ T-cell output is reduced with aging, which is not solely due to reduced HSC functionality (Fig. 1). Thymic involution, which is the progressive decrease in size of the thymus, contributes to a strong reduction of naïve T-cell production. In humans, by the age of 70, thymus activity is completely lost [24] and the maintenance of the naïve T-cell pool is made through homeostatic proliferation [25]. In contrast, in mice, thymic functionality is relatively well maintained with age [26]. It highlights species differences in immune responses. Nevertheless, in both mice and humans, the TCR repertoire of naïve T cells in the circulation is reduced with aging [27–29]. Homeostatic proliferation favors the accumulation of particular clones in the naïve T-cell pool further contributing to reduced diversity of the TCR repertoire. In this context, chronic infections, such as cytomegalovirus infection, lead to the alteration of the TCR repertoire with an accumulation of low-affinity TCR clones [30]. In this context, aged mice fail to properly respond to influenza infection due to the loss of the TCR repertoire diversity [31]. Therefore, with the reduction of the TCR repertoire, the ability of naive T cells to respond to new incoming antigens is reduced, which leads to an immunocompromised state. Of note, multiple strategies to improve thymic output have been proposed [32]. For instance, recombinant interleukin (IL)-7 administration has been shown to improve the naïve T-cell pool [33]. In addition to reduced naïve T-cell repertoire diversity, aged naïve T cells differentiate toward a memory-like phenotype (so-called virtual memory) with impaired functionality. This process has been shown to be driven by homeostatic proliferation in the lymphopenic environment [34, 35]. Of note, these cells maintain IL-15 responsiveness [36]. Notably, the formation of virtual memory T cells can be controlled by TCR stimulation since TCR transgenic mouse strain does not have this kind of population [37]. The ability of both naïve T cells and virtual memory T cells to respond to infection and develop memory T cells after infection is reduced in aged mice [28, 38, 39]. Strikingly, aged T cells also display an exhausted phenotype, a specialized differentiation state characterized by declined proliferative capacity and effector function and sustained expression of inhibitory receptors [40], and express the transcription factor Thymocyte selection-associated HMG Box (TOX) [41]. In addition, they express multiple inhibitory checkpoint receptors including programmed cell death protein-1 (PD-1), T-cell immunoglobulin and mucin-domain containing-3, and lymphocyte activation gene-3 (Lag3) [28]. Moreover, they highly upregulate the expression of Granzyme K which might further drive a systemic aging phenotype [41]. In this context, aged T cells also display characteristics of senescent T cells, including reduced expression of costimulatory molecules and upregulated Killer cell lectin-like receptor subfamily G member 1 (KLRG1) or CD57 [42]. They also display altered chromatin architecture, accumulate DNA damage, and reduce telomere length [43], which can be inherited in HSCs [44–46]. Interestingly, chronic infection might also contribute to the induction of senescence in T cells [47]. Of note, aged T cells upregulate the production of several proinflammatory cytokines, including tumor necrosis factor (TNF), IL-6, and osteopontin, which further contributes to the establishment of an aging phenotype in tissues and organs. Taken together, aged T cells develop an exhausted/senescent phenotype that correlates with reduced functionality, which results in an immunocompromised state. Among the CD4^+^ T-cell compartment, regulatory T cells (Tregs) accumulate with age [48, 49]. Interestingly, Tregs undergo increased senescence as compared to effector T cells [50]. In addition, compared to young individuals, Tregs from aged individuals are more activated, and effector T cells from aged individuals highly upregulate proinflammatory cytokines and cytotoxicity [51]. Strikingly, the impairment of the aged CD4^+^ T-cell compartment has also been associated with a decreased humoral response [52], which might further drive an immunocompromised state.

Taken together, the impaired functionality of CD4^+^ and CD8^+^ T cells along with innate immune cells contributes to the development of inflammaging, a hallmark of aging [53]. This process is described as permanent low-grade inflammation and is a critical risk factor toward morbidity and mortality in elderlies. Of note, proinflammatory cytokines, including IL-6 and C-reactive protein, are used as markers of inflammaging. IL-6 and TNF have also been associated with age-related co-morbidities including cardiovascular disease in frailty [54]. In addition, Type I interferons are involved in cognitive decline during aging, further highlighting the deleterious effect of inflammaging [55]. In this context, T cells have been recognized as drivers of inflammaging through the secretion of proinflammatory cytokines such as TNF and IL-6 and the neutralization of TNF has been shown to reverse a premature aging phenotype in T lymphocytes [56]. However, it remains unclear whether anti-inflammatory agents can ameliorate aging and the efficacy and safety of this type of strategy remain to be determined. Furthermore, senescent T cells may drive inflammaging via other cytokines produced as a result of senescence-associated secretory phenotype. In this context, reduced immune cell function might also lead to the reduction of senescent cell clearance which might further facilitate the inflammaging program [57].

## Metabolism of T cell aging

**Figure 2:**
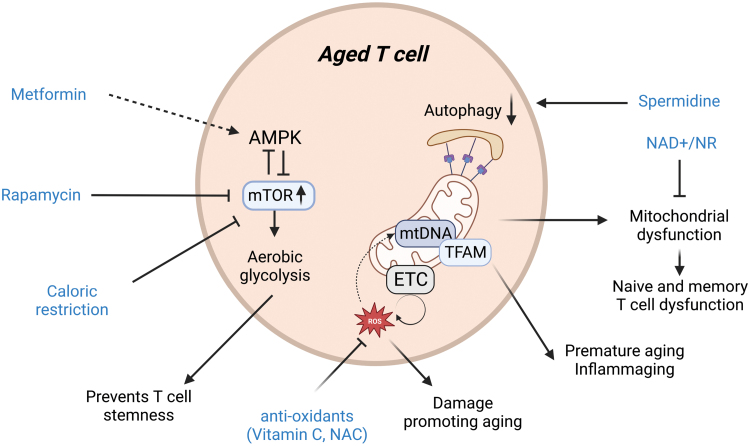
mTOR pathway is activated and maintained in aged T cells, leading to high glycolytic metabolic profile. This pathway presents multiple therapeutic opportunities such as targeting AMPK with metformin or mTOR directly with rapamycin. The mitochondrial machinery is also dysfunctional in aged T cells due to impaired mitochondrial biogenesis, accumulation of damages and declined autophagy activity. NAD^+^/NR supplementation can fuel many redox reactions and improve mitochondrial function. Antioxidants like vitamin E or NAC can prevent mitochondrial ROS mediated damages and improve the cellular redox balance. Reduced autophagy activity in aged T cells can be reactivated by spermidine to promote memory T cells.

Metabolism is known to be critical to support CD4^+^ T-cell function and differentiation; however, aged CD4^+^ T cells present an altered metabolic reprogramming characterized by a compromised ability to upregulate OXPHOS and glycolysis to support their function [58]. A recent work showed that T cells from elderly donors display disrupted lipid metabolism, which may contribute to a blunted T-cell immune response by perturbing T-cell proliferation and susceptibility to the apoptosis pathway [59]. Elevated steady-state glycolytic metabolism is another well-known feature of aged T cells (Fig. 2). This increased glycolytic profile is likely controlled by the increased basal level and activation state of mTOR protein as well as the level of ribosomal protein S6 and its phosphorylation [60]. Therefore, elevated glycolytic activity in CD8^+^ T cells might reduce the life span of naïve and memory T cells [61]. It has been well reported that mTOR is a crucial lifespan regulator [62, 63] by controlling cell proliferation, autophagy, mitochondrial fitness, metabolic reprogramming, and cellular senescence [64]. mTOR is also known for its role in nutrient sensing since mTOR orchestrates anabolic metabolism when mTOR ensures that cells have sufficient building blocks and energy. In regard to amino acid sensing process, amino acids induce the activation of Rag GTPases, at the surface of lysosomes, leading to their binding to the regulatory associated protein of mTOR (RAPTOR) and promoting mTOR recruitment [65, 66]. Additionally, the protein kinase AMPK senses AMP levels upon nutrient starvation or low oxygen and negatively regulates mTOR by phosphorylating tuberin [67] and RAPTOR [68]. A key hallmark of aging is dysregulated nutrient sensing and elevated mTOR basal level. Given the central role of mTOR in cellular functions, it is likely that mTOR also impacts other aging hallmarks like the dysfunction of the protein synthesis machinery [69, 70]. In addition, aging cells also present defective protein quality control and degradation machinery, like autophagy, which results in the accumulation of damaged proteins and organelles [71]. A direct link between constitutive mTOR activation and disturbed autophagy was made in fibroblasts [72] and approaches aiming to restore or improve autophagy machinery are nowadays investigated to promote lifespan [73]. In neutrophils, it was shown that phosphoinositide 3-kinase, an upstream enzyme of the mTOR pathway, was constitutively active in old subjects and caused aberrant neutrophil migration. Inhibition of mTOR reverses the aged phenotype and improves the accuracy and regulations of migration [20]. In addition to the mTOR pathway, in aged senescent memory T cells, the MAPK cascade is hyperphosphorylated, causing perturbations in T cell’s proliferative capacity, calcium flux, and cytokine production [74]. The stress-induced proteins called sestrins bind to the MAP kinases extracellular signal-regulated kinase, c-Jun NH_2_-terminal kinase, and p38 to form an immune–inhibitory complex and disruption of those complexes restores T-cell function.

Aged naive T cells present signs of mitochondrial dysfunction characterized by impaired mitochondrial biogenesis upon activation with smaller mitochondria and lower spare respiratory capacity [75]. The loss of respiratory capacity is driven by a reduced expression of genes encoding the electron transport chain [76]. One-carbon metabolism is also perturbated and the proliferation and activation of aged T cells could be rescued by supplementation of the one-carbon metabolites, including formate and glycine. Ceramide metabolism in aged mitochondria has also been reported to be imbalanced. Activated aged T cells have elevated C14/C16 ceramide levels due to the action of the ceramide synthase 6 [77], which in turn leads to impaired mitophagy machinery and mitochondrial dysfunction by inhibiting protein kinase A. Further evidence shows that mitochondrial dysfunction and lack of autophagy contribute to the establishment of the aged phenotype in T cells [78]. In addition, deletion of key autophagy proteins leads to a loss of memory T cells [79, 80]. Autophagy enhancement by metformin treatment resulted in mitochondrial shape normalization and reversed the inflammaging phenotype in another study [81]. Moreover, spermidine treatment improved memory T-cell formation of aged mice after vaccination [80]. Strikingly, aged naïve T cells display reduced autophagy flux, which might contribute to the accumulation of damaged mitochondria. The accumulation of mitochondrial DNA (mtDNA) mutations also contributes to aging, even in the absence of ROS or oxidative stress. Interestingly, mtDNA mutations are a driver of aging as highlighted by publications using a deficient version of the proofreading mtDNA polymerase in mammals to promote mtDNA mutations [82, 83]. Strikingly, mitochondrial dysfunction in T cells caused by genetic ablation of mitochondrial transcription factor A has been recently shown to be a driver of premature aging [56]. Of note, through aging, dysfunction of mitochondria has been linked to neurological disorders. For instance, mutations in proteins involved in mitophagy (the safeguard of mitochondrial health), including PTEN-induced kinase 1, Park2, and leucin-rich repeat kinase 2, lead to mitochondrial dysfunction and the development of Parkinson’s disease (PD) [84–87]. In addition, mitochondrial dysfunction is also associated with the development of Alzheimer’s disease [88]. Interestingly, despite that immune defects have been observed in those patients with PD or Alzheimer’s disease, it remains largely unclear whether the decline of mitochondrial fitness and dynamics in T cells and other immune cells in those patients is the underlying mechanism for impaired immune responses. Of note, oxidative stress is a critical factor involved in the development of aging and is linked to many diseases associated with aging [89]. In this context, mitochondrial metabolism is known to be a source of ROS that in certain conditions functions as a signaling molecule [90, 91]. However, ROS accumulated to toxic levels is deleterious to cells. Therefore, failure to maintain mitochondrial fitness might lead to mitochondrial ROS generation which might in turn drive aging processes. Notably, the loss of autophagy and mitophagy activity in tumor-infiltrating T lymphocytes leads to mitochondrial dysfunction associated with increased mitochondrial ROS production [92]. Taken together, impaired mitochondrial metabolism drives the development of aging phenotypes and contributes to disease development in the advanced aged.

Some homeostatic signaling pathways, linked to metabolism, are also dysregulated in aged T cells. IL-7 signaling is known to decline in aged mice and humans [93]. IL-7 is crucial for the survival and pool maintenance of naïve T cells by driving the expression of anti-apoptotic factors such as B cell lymphoma-2, but also plays a role in the differentiation into memory T cells [94–96]. IL-7 signaling has also been linked to metabolic fitness since it triggers the upregulation of aquaporin 9 in memory T cells to import glycerol and drives triglyceride synthesis and stockage [97]. This process sustains ATP levels and promotes memory CD8^+^ T-cell survival. Defects in the different messengers involved in signal transduction upon TCR engagement have been observed in aged T cells [98]. Calcium flux has been reported to be diminished in T cells from aged mice following activation [99, 100]. One could expect the nuclear factor of activated T cell signaling to be impacted by those calcium flux dysregulation, however, the underlying molecular mechanisms are still unclear and under investigation [101].

## Modulation of metabolism to improve the response of the aged immune system

As highlighted in the previous section, T cells undergo profound metabolic impairment with aging. Thus, targeting and manipulating those metabolic pathways might be promising strategies to improve function and immune response in aged T cells (Fig. 2). For example, pharmacological inhibition of glycolysis by 2-deoxy­glucose has been shown to result in increased cell longevity and induction of memory phenotype [102]. Conversely, enforcing glycolytic metabolism by genetic overexpression of phosphoglycerate mutase 1 (*Pgam1*) leads to decreased survival and skews toward a terminally differentiated state. mTOR axis is another promising target to reverse aging phenotype as a result of metabolic reprogramming. mTOR’s most common inhibitor rapamycin is currently studied in different cell types to prevent or reverse age-related hallmarks. More specifically, rapamycin has been shown to enhance the *in vivo* regenerative ability of HSCs from old mice and improve the capacity of those mice to mount an effective immune response to viral infection by boosting life span and the ability of self-renewal and hematopoiesis in aged HSCs [103]. In CD8^+^ T cells, rapamycin treatment can promote the differentiation into long-lived memory cells and improve both the quantity and quality of those cells without impacting the effector phase of the immune response [5, 6]. In support of these studies, clinical trials conducted in elderly volunteers highlight that mTOR inhibition boosts the immune response to seasonal influenza vaccination by increasing antibody levels and dampening expression of PD-1 in T cells, and reduces the overall rate of infection [104, 105]. mTOR can also be blunted by activating AMPK with metformin. Metformin action has been studied extensively and consistently shown anti-aging benefits [106]. In T cells, metformin treatment has been shown to reduce apoptosis and increase the formation of memory cells [107]. However, the exact mechanisms of actions remain unclear and could be attributed to other pathways than inhibition of mTOR like decreased IGF-1 signaling or inhibition of mitochondrial complex 1.

Mitochondrial fitness could also be modulated to improve the immune response of aged T cells. Nicotinamide adenine dinucleotide (NAD^+^) is a metabolite involved in many redox reactions fueling diverse metabolic pathways. However, systemic levels of NAD^+^ drop with age. Supplementation with NAD^+^ was shown, in multiple cell types, to promote mitochondrial function [108]. Treatment with nicotinamide riboside (NR), a precursor of NAD^+^, induces mitochondrial unfolded protein response and synthesis of prohibitin proteins, which are involved in multiple mitochondrial functions [109], and rejuvenates stem cells in aged mice [108, 110]. In immune cells, NR treatment was shown to improve mitochondrial fitness in HSCs and CD8^+^ T cells in both aging and exhaustion contexts [92, 111]. In addition, the induction of the cellular recycling pathway may also improve the function of aged T cells. For instance, spermidine, an autophagy inducer, has been shown to boost memory CD8^+^ T-cell formation following vaccination in aged mice [80]. Of note, spermidine is a polyamine compound that has been previously suggested to prevent aging processes [112]. For instance, mice fed with spermidine display increased lifespan and spermidine protects cardiovascular function [113]. Interestingly, in CD8^+^ T cells, urolithin A, a mitophagy inducer compound, promotes stem cell memory T cells in tumor context via the maintenance of mitochondrial function [114]. In this context, compounds able to induce mitophagy such as urolithin A may boost aged T-cell function.

Since ROS is involved in promoting aging in immune cells, modulation of the cellular redox balance represents an attractive approach to prevent immune cell aging. Approaches such as vitamin E supplementation has been shown to enhance T-cell differentiation and function in aged mice and rats [115, 116]. In elderly humans, Meydani *et al*. reported that exogenous apport of vitamin E increased, *ex vivo*, lymphocyte proliferation and IL-2 production, and reduced the production of the immunosuppressive prostaglandin E2 [115]. In this context, improving the antioxidant machinery to ameliorate the immune system aging becomes an interesting approach. Strikingly, the antioxidant machinery is decreased with age [117]. Of note, NRF2 is a major transcription factor involved in the regulation of the cellular antioxidant machinery and its activity has been suggested to be decreased with aging. Therefore, modulation of NRF2 activity may represent an attractive strategy to improve the cellular redox balance during aging. In addition, supplementation of N-acetyl cysteine (NAC) and vitamin C favors memory T-cell formation in aged mice, further suggesting that antioxidant therapy may promote immune functionality [118]. Calorie restriction (CR) delays cell senescence and prolongs lifespan in mice and humans [119, 120]. CR improves fatty acid metabolism [121] and delays T-cell senescence in primates by preserving T-cell repertoire diversity and improving T-cell function [122]. Recent studies have also shown that CR also decreases senescence-associated T cells in aged mice, further supporting its use in therapeutic settings [123]. Overall further investigations are necessary to better understand which metabolic pathways should be targeted and how to target them in a safe way to prevent unwanted effects.

## Conclusion

The immune response undergoes profound changes with aging. As discussed in this review, these changes lead to a loss of protection against pathogenic threats but also the establishment of an environment that is detrimental. In this context, understanding how the immune response is altered with age and the underlying cause represents a great challenge in improving immune functionality during therapies. Recent advances have highlighted the importance of metabolic regulations in orchestrating T-cell behavior. Interestingly, metabolic processes are also altered in aging, which prevents proper immune cell functionality. In this context, metabolic interventions might provide new therapeutic avenues to improve therapies for the elderlies including vaccination and cancer treatments. Several axes are already being studied in their ability to improve human health including antioxidant therapy, diet, autophagy inducers, and glycolysis inhibitors.
